# The frequency and risk factors of major complications after thermal ablation of liver tumours in 2,084 ablation sessions

**DOI:** 10.3389/fsurg.2022.1010043

**Published:** 2022-09-15

**Authors:** Qiannan Huang, Mengya Pang, Qingjing Zeng, Xuqi He, Rongqin Zheng, Mian Ge, Kai Li

**Affiliations:** ^1^ Department of Ultrasound, Guangdong Key Laboratory of Liver Disease Research, The Third Affiliated Hospital of Sun Yat-sen University, Guangzhou, China; ^2^ Department of Anesthesiology, The Third Affiliated Hospital of Sun Yat-sen University, Guangzhou, China

**Keywords:** thermal ablation, liver tumours, major complications, ultrasound, risk factors

## Abstract

**Background:**

To assess the frequency of major complications after thermal ablation of liver tumours and to determine risk factors for adverse events.

**Methods:**

A retrospective study was conducted between January 2015 and January 2021. A total of 2,084 thermal ablation sessions in 1,592 patients with primary and metastatic liver tumours were evaluated. The frequency of major complications was evaluated according to the Society of Interventional Radiology Standards, and putative predictors of adverse events were analysed using simple and multivariate logistic regression.

**Results:**

Thermal ablation-related mortality was 0.1% (2/2,084), with an overall major complication rate of 5.6% (117/2,084). The most frequent major complication was symptomatic pleural effusion (2.9%, 60/2,084). Multivariate logistic regression analysis revealed that a total maximum diameter of lesions >3 cm, microwave ablation (MWA) and MWA combined with radiofrequency ablation, intrahepatic cholangiocarcinoma and postoperative systemic inflammatory response syndrome were independent prognostic factors for major complications.

**Conclusions:**

Thermal ablation of liver tumours is a safe procedure with an acceptable incidence of major complications. The risk factors identified in this study will help to stratify high-risk patients.

## Highlights

1. Thermal ablation of liver tumours is a safe procedure with an acceptable incidence of major complications.
2. In 2,084 thermal ablation sessions, we found ablation-related mortality was 0.1% (2/2,084), with an overall major complication rate of 5.6% (117/2,084).
3. A total maximum diameter of lesions >3 cm, MWA and MWA combined with RFA, ICC and postoperative SIRS were found to be independent prognostic factors for major complications.

## Introduction

Liver malignancies are the most commonly diagnosed cancers and are the fourth leading cause of cancer-related deaths worldwide (1, 2). Percutaneous thermal ablation, including radiofrequency ablation (RFA) and microwave ablation (MWA), has been widely accepted as a curative and minimally invasive treatment for patients with hepatic malignancies, including hepatocellular carcinoma (HCC) and liver metastases (3–5). Thermal ablation has shown comparable therapeutic effects after hepatic resection in patients with very early-stage HCC (6, 7). Compared with traditional surgical treatments, thermal ablation of tumours has a relatively low incidence of complications, and major complications caused by thermal ablation are reported around 2%–7.9% (8–15). Although thermal ablation is considered relatively safe and minimally invasive, it can induce severe, life-threatening complications, such as hepatic failure, intraperitoneal bleeding, hepatic abscess, bile duct injury and gastrointestinal perforation (16, 17). A better understanding of these complications is the key to successful utilization of thermal ablation as a treatment strategy. Therefore, the purpose of this retrospective study was to assess the frequency of major complications after percutaneous thermal ablation and evaluate risk factors that may account for them.

## Materials and methods

### Patients

**Figure 1:**
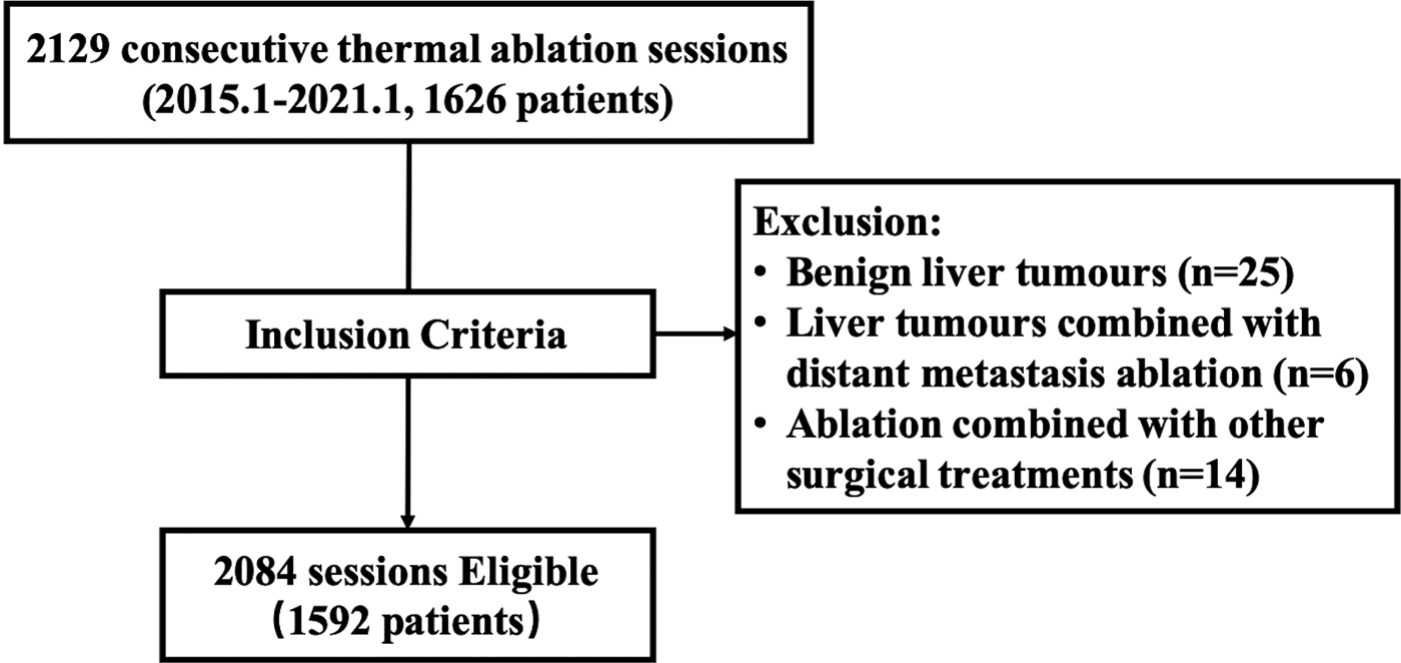
Flowchart of thermal ablation selection.

This retrospective study followed the Declaration of Helsinki ethical principles and was approved by the Ethics Committee of the Third Affiliated Hospital of Sun Yat-sen University. A total of 2,129 consecutive ultrasound-guided thermal ablation sessions in 1,626 patients with hepatic cancer between January 2015 and January 2021 were screened for study eligibility. Patients were from the Third Affiliated Hospital of Sun Yat-sen University. The diagnosis of liver malignancies was confirmed on the basis of pathology or the non-invasive criteria defined by the American Association for the Study of Liver Disease: arterial hyperenhancement with venous or delayed-phase washout as seen on imaging (18). Ablations for benign liver lesions (*n* = 25), liver tumours combined with distant metastasis ablation, including portal vein embolus, spleen, adnexal, adrenal gland, and abdominal metastasis (*n* = 6), and ablation combined with other surgical treatments during the patient's hospitalization, including transcatheter arterial chemoembolization (*n* = 4), liver transplantation, proctectomy, cholecystectomy or thyroidectomy (*n* = 6) and others (*n* = 4), were excluded (Figure 1). The remaining 2,084 consecutive thermal ablations were then analysed. Baseline characteristics of patients and perioperative laboratory, clinical and imaging data were acquired from the computerized databases of our institution.

### Instruments

In this study, thermal ablation system included RFA and MWA. A cooled-tip RFA system (Covidien, Mansfield, MA, USA) with an internally cooled electrode was used. A 2,450 MHz microwave generator (Kangyou, Nanjing, China) with an internally cooled microwave antenna was used. A MyLab Calss or MyLab Twice ultrasound machines (Esoate, Genoa, Italy) with a CA431(frequency: 4–10 MHz) or CA541 (frequency: 1–8 MHz) convex array probe was used during thermal ablation.

### Thermal ablation

All thermal ablation procedures, including RFA and MWA, were performed under ultrasound guidance by senior interventional sonographers with at least six years of experience performing this procedure. Ablation was performed under tracheal general anaesthesia in the operating room. RFA or MWA was selected according to the tumour size, location and patient status. For lesions directly adjacent to critical organs and structures, such as major hepatic vessel, diaphragm, gastrointestinal tract, gallbladder, and major intrahepatic bile duct, or in difficult puncture locations, RFA was preferred. In some circumstances, including for tumors >3 cm in maximum diameter, distance to critical organs and structures >5 mm, or patients with abnormal coagulation function, MWA was preferred. Generally, the RF generator was set in impedance mode with a maximum output. Each insertion of an RF electrode took approximately 12 min. The MW generator was set at 60 watts and maintained for 6 min in each MW antenna insertion. All tumour ablation procedures were aimed for complete ablation. Some characteristics of lesions would increase the difficulty of the ablation, therefore, auxiliary methods were used. When the visualization of lesions was impeded by the diaphragm or gas in the lungs, artificial pleural fluid was employed. When lesions were adjacent to the gastrointestinal tract and gallbladder, artificial ascites was employed. When lesions were adjacent to the bile duct, percutaneous intraductal perfusion with chilled saline was employed to cool and protect the bile duct.

### Major complications

Major complications related to thermal ablation were recorded and evaluated using all available medical records, including imaging reports. Major complications were defined according to the Society of Interventional Radiology (SIR) Standards of Practice Committee classification (19). The definition of major complications is an event that led to substantial morbidity and disability (e.g., the unexpected loss of an organ), that increased the level of care required, resulted in hospital admission, or substantially lengthened the hospital stay (SIR classifications C–E). This includes any case in which a blood transfusion or interventional drainage procedure was required (20). Deaths related to thermal ablation were also recorded.

### Definitions of systemic inflammatory response syndrome (SIRS)

Postoperative SIRS was included as a variable in this study if it occurred within seven days after ablation and before the development of any major complications. SIRS was defined as the presence of any two or more of the following: body temperature of >38 °C or of <36 °C; a heart rate of >90 beats/min; respiratory rate of >20 breaths/min or a PaCO2 of <32 mmHg; white blood cell count of >12 × 10^9^/L or of <4 × 10^9^/L; or the presence of 10% immature neutrophils.

### Statistical analysis

Statistical analysis was performed using SPSS 25.0 for Mac OS (SPSS Inc., Chicago, IL, USA). Categorical variables were expressed as numbers and compared using the Chi-squared (*χ*^2^) test. Independent continuous variables are expressed as the median (range) and were compared using the Mann–Whitney U test. Binary logistic regression was used to evaluate possible predictors of major complications. Variables of interest that were identified in a simple logistic regression analysis (*p *< 0.1) were further analysed in a multivariate model. *p* < 0.05 was considered to be statistically significant.

## Results

### Enrollment

A total of 2,084 consecutive thermal ablations in 1,592 patients were analysed. The baseline characteristics are shown in Table 1.

**Table 1:** Baseline characteristics of 1,592 patients in 2,084 ablation sessions.

Characteristic	Value
Age, years (median, range)	56 (21–87)
Gender (Male/Female)	1,789/295
Tumour type (HCC/ICC/Metastasis)	19,59/34/91
ASA score (1/2/3)	354/1,400/330
Hypertension (No/Yes)	1,465/619
Diabetes (No/Yes)	1,546/538
HGB (g/L)	138 (48–186)
INR	1.07 (0.78–2.55)
PLT (×10^9^/L)	132 (20–478)
ALB (g/L)	41 (20–56)
TBIL (umol/L)	12.6 (2.3–172.5)
Child–Pugh score (A/B/C)	1,946/132/6
Portal hypertension (No/Yes)	1,026/1,058
Cirrhosis (No/Yes)	463/1,621
Etiology of the liver disease (viral hepatitis/others/none)	1,921/25/138
No. of tumours (Solitary/Multifocal)	1,349/735
Total maximum diameter of the lesions (≤30 mm/>30 mm)	1,351/733
Thermal ablation method used (RFA/MWA/RFA + MWA)	1,111/924/49
Requirement of auxiliary methods for ablation (No/Yes)	1,081/1,003
Postoperative SIRS (No/Yes)	1,784/300

HCC, hepatocellular carcinoma; ICC, intrahepatic cholangiocarcinoma; ASA, American Society of Anesthesiologists; HGB, Haemoglobin; INR, International normalized ratio; PLT, Platelet; ALB, Albumin; TBIL, Total bilirubin; RFA, Radiofrequency ablation; MWA, Microwave ablation; Etiology of the liver disease, viral hepatitis includes Hepatitis B and C; others includes fatty liver, alcoholic liver disease, autoimmune liver disease, Budd-Chiari syndrome, cholestasis liver diseases, primary biliary cirrhosis and liver fluck disease. Total maximum diameter of the lesions: The sum of the maximum diameters of all lesions undergoing ablation; Requirement of auxiliary methods for ablation included artificial pleural fluid and ascites, laparotomy, laparoscopy and percutaneous intraductal chilled saline perfusion. SIRS, systemic inflammatory response syndrome.

### Mortality

Two deaths (0.1%, 2/2,084) were related to liver thermal ablation (Table 2). One patient died of multiple organ dysfunction syndrome 16 days after ablation. The other one occurred major hemorrhage in the abdominal cavity within 3 days after ablation, then developed hemorrhagic shock, and finally, died one month after ablation.

**Table 2:** Major complications after 2,084 thermal ablation sessions.

Major complications	No.	Therapy
Multiple organ dysfunction syndrome	1	ICU (death)
Haemorrhage in abdominal cavity	2	One in ICU (death), one needed blood transfusion
Haemorrhage in pleural cavity	2	Surgery
Transient cardiac problems	1	ICU
Liver abscess	25	Ultrasound-guided drainage
Biliary fistula	1	Ultrasound-guided drainage
Ascites requiring treatment	14	Ultrasound-guided drainage
Biloma	2	Ultrasound-guided drainage
Symptomatic pleural effusion	60	Thoracentesis
Ablation zone pneumatosis	5	Ultrasound-guided aspiration
Upper gastrointestinal bleeding	1	Blood transfusion
Secondary thrombocytopenia	1	Platelet transfusion
Pulmonary infection, Transient respiratory failure	2	Broad-spectrum antibiotics

ICU, Intensive care unit.

### Major complications

Major perioperative complications are shown in Table 2. The major complication rate was 5.6% (117/2,084). In addition to the aforementioned fatal complications, the major complications included the following: pleural effusion, which was the most frequent major complication requiring thoracentesis (51.3%, 60/117); transient cardiac problems requiring intensive care unit admission (0.9%, 1/117); major haemorrhage in the abdominal cavity (0.9%, 1/117), upper gastrointestinal bleeding (0.9%, 1/117) and secondary thrombocytopenia (0.9%, 1/117) requiring blood or platelet transfusion; major haemorrhage in the pleural cavity requiring surgical repair (1.7%, 2/117); liver abscess (21.4%, 25/117), biliary fistula (0.9%, 1/117), ascites requiring treatment (11.9%, 14/117) and biloma (1.7%, 2/117) requiring ultrasound-guided drainage; ablation zone pneumatosis requiring ultrasound-guided aspiration (4.3%, 5/117); and pulmonary infection resulting in transient respiratory failure and requiring broad-spectrum antibiotics (1.7%, 2/117).

The median postoperative hospital stay was 4 days (1–116) and was significantly longer in patients who developed major complications, with a median stay of 12 days (2–116) (*p *< 0.001).

### Risk factors for major complications

Albumin and total bilirubin were excluded from the analysed variables, as they were already included in the Child–Pugh scoring system that was used to assess liver disease prognosis. A univariate analysis showed that significant predictors of major complications were: (i) tumour type (*p *= 0.001), (ii) American Society of Anesthesiologists (ASA) score (*p* = 0.011), (iii) diabetes (*p *= 0.010), (iv) haemoglobin levels (HGB) (*p *= 0.006), (v) platelet counts (PLT) (*p *< 0.001), (vi) Child–Pugh score (*p *= 0.006), (vii) etiology of the liver disease (*p *= 0.016), (viii) no. of tumours (*p *= 0.002), (ix) total maximum diameter of the lesions (*p *< 0.001), (x) thermal ablation method used (*p *< 0.001), (xi) requirement of auxiliary methods for ablation (*p *= 0.009) and (xii) Postoperative SIRS (*p *< 0.001)(Table 3).

**Table 3:** Univariable logistic regression analyses of risk factors for major complications.

Variables	major complication (No)	major complication (Yes)	*p* value
Age, years (median, range)	56 (23–87)	58 (21–87)	0.256
Gender (Male/Female)	1,691/276	98/19	0.506
Tumour type (HCC/ICC/Metastasis)	1,857/27/83	102/7/8	0.001^a^
ASA score (1/2/3)	341/1,325/301	13/75/29	0.011^a^
Hypertension (No/Yes)	1,376/591	89/28	0.160
Diabetes (No/Yes)	1,471/496	75/42	0.010^a^
HGB (≥130/<130) (g/l)	1,302/665	63/54	0.006^a^
INR (≤1.5/>1.5)	1,926/41	112/5	0.180
PLT (≥100/<100) (×10^9^/l)	1,387/580	63/54	<0.001^a^
Child–Pugh score (A/B–C)	1,844/123	102/15	0.006^a^
Portal hypertension (No/Yes)	974/993	52/65	0.286
Cirrhosis (No/Yes)	435/1,532	28/89	0.646
Etiology of the liver disease (viral hepatitis/others/none)	1,821/22/124	100/3/14	0.016^a^
No. of tumours (Solitary/Multifocal)	1,289/678	60/57	0.002^a^
Total maximum diameter of the lesions (≤30 mm/>30 mm)	1,310/657	41/76	<0.001^a^
Thermal ablation method used (RFA/MWA/RFA + MWA)	1,063/864/40	48/60/9	<0.001^a^
Requirement of auxiliary methods for ablation (No/Yes)	1,034/933	47/70	0.009^a^
Postoperative SIRS (No/Yes)	1,723/244	61/56	<0.001^a^

^a^ Statistically significant.

**Figure 2:**
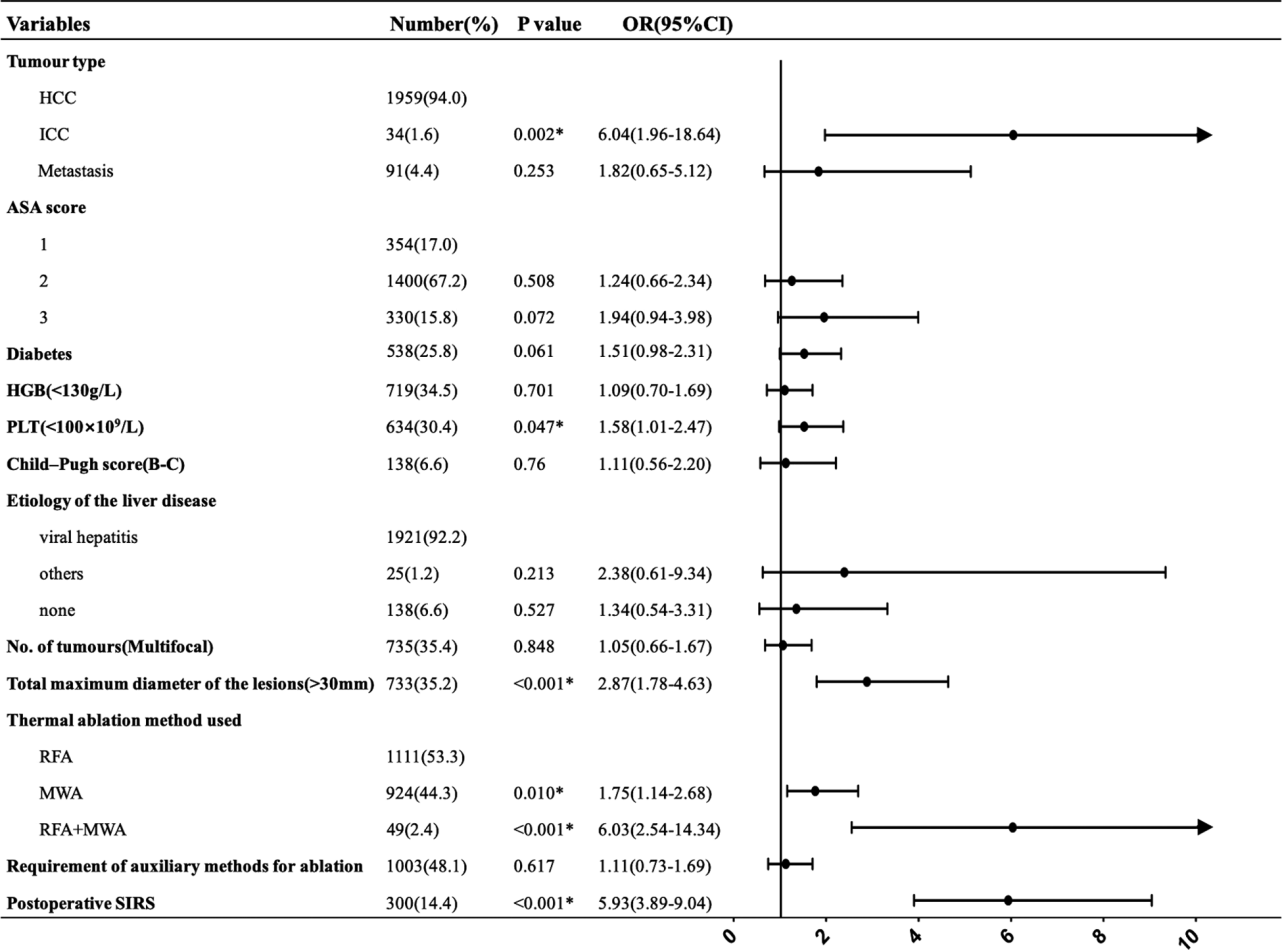
Multivariate logistic regression analyses of risk factors for major complications. *Statistically significant.

After multivariate analysis, intrahepatic cholangiocarcinoma (ICC) (*p *= 0.002); total maximum diameter of the lesions >3 cm (*p *< 0.001); thermal ablation method used, including MWA (*p *= 0.010) and RFA plus MWA (*p *< 0.001); and postoperative SIRS (*p *< 0.001) were found to be independent prognostic factors. PLT count <100 × 10^9^/L was statistically significant in increasing the incidence of major complications(*p *= 0.047). Figure 2 shows multivariate logistic regression analyses of risk factors for major complications. ICC (OR = 6.04), a total maximum diameter of the lesions >3 cm (OR = 2.87) and postoperative SIRS (OR = 5.93) had a greater probability of developing major complications. Compared with RFA, MWA and RFA + MWA increased the major complication risk 1.75-fold and 6.03-fold, respectively. Furthermore, we found a 1.58-fold increased risk of developing major complications in patients with a PLT count <100 × 10^9^/L.

## Discussion

In this study of 2,084 ablation sessions performed over 5 years, we showed that thermal ablation is a safe treatment for liver malignancies, with a mortality rate of 0.1% (2/2,084) and an overall major complication rate of 5.6% (117/2,084), which is similar to the results of previous studies (8–15). Table 4 showed the incidence of common major complications between previous studies (8–17) and this study. Most of the common major complications in this study were similar or even lower than in previous studies. In this study, the most frequent major complication was symptomatic pleural effusion (2.9%, 60/2,084), which we found at higher rates than what has been reported in previous studies (13, 14, 16, 21). The reason for this discrepancy might be selection bias of tumours. Our previous study concluded that the frequency of subphrenic tumours was 24% (22), a higher frequency than other studies had previously reported 6.3%–13.5% (23, 24). Thermal ablation of liver tumours abutting the diaphragm poses a risk of diaphragmatic injury, and symptoms range from mild, such as pleural effusion and right shoulder pain, to severe, such as diaphragmatic perforation (23, 25). Mild diaphragmatic injury is self-limiting and asymptomatic pleural effusion can resolve on its own. However, thoracentesis or diuresis is required if the patient experiences dyspnea or chest tightness. In addition, when tumours were located in the subphrenic region, artificial pleural effusion was used to improve the sonic window of tumours, to visualize and ablate the tumour completely and, to decrease the damage to diaphragm. The thoracic drainage tube was kept in for several days to observe the drainage of the pleural effusion. There were no diaphragmatic perforations in this study, and all symptomatic pleural effusions were significantly relieved after 3–5 days of thoracic drainage.

**Table 4:** Incidence of common major complications between previous studies and this study.

Major complications	Incidence in previous studies	Incidence in this study
Haemorrhage in abdominal cavity	0.5%–4.9%	0.1% (2/2,084)
Haemorrhage in pleural cavity	0.1%–0.3%	0.1% (2/2,084)
Liver abscess	0.35%–1.6%	1.2% (25–2,084)
Gastrointestinal perforation	0.06%–0.4%	0%
Biliary fistula	0.1%–1%	0.05% (1/2,084)
Biloma	0.09%–1.3%	0.1% (2/2,084)
Ascites requiring treatment	0.2%–0.8%	0.7% (14/2,084)
Symptomatic pleural effusion	0.8%–2.1%	2.9% (60/2,084)
Liver dysfunction (including upper gastrointestinal bleeding)	0.1%–1.2%	0.05% (1/2,084)

The results showed that the only independent predictive factors for major complications were: total maximum diameter of the lesions >3 cm; thermal ablation method used, including MWA and MWA + RFA; and ICC and postoperative SIRS. PLT counts <100 × 10^9^/L, although statistically significant, appeared to be a trend to increase the incidence of major complications.

The present study suggested that the ablation of larger size tumours leads to more major complications, which is consistent with the published literature (9). It is conceivable that larger tumours require more ablation treatments and require the administration of higher ablation energy. Single ablation usually fails to achieve sufficient coverage of larger tumours, and multiple overlapping ablations are necessary (26), which may increase the complications caused by puncture. A larger ablated zone may have a greater impact on the liver function reserve. For tumours larger than 3 cm, MWA is preferred due to its high thermal efficiency, higher capability for coagulation of blood vessels and faster ablation time (27). When large tumours were located adjacent to vital organs, such as the gallbladder and gastrointestinal tract, we combined RFA on the side of the tumour close to vital organs to completely ablate the tumour while reducing damage to vital organs. Although the results showed that when the total maximum diameter of the lesions was >3 cm and thermal ablation methods including MWA and MWA + RFA, increased the occurrence of major complications, half of the complications were symptomatic pleural effusion, and the thermal damage caused by ablation was relatively limited.

Compared with HCC, ICC is more aggressive and requires a larger ablative range to avoid local tumour progression (28), which may damage adjacent structures. In addition, ICC usually causes obstruction of the biliary tract by inhibiting adequate drainage of bile leading to increased probability of liver abscess due to retrograde infection. Su et al. (29) reported that 42.8% of patients with ICC developed abscesses after ablation procedures due to the increased risk of ascending biliary infection. In this study, the incidence of liver abscess in patients with ICC was 4.4% (4/91), higher than the incidence in patients with HCC (1.1%, 21/1,959).

Although the results showed that *p* value of patients with PLT count <100 × 10^9^/L was less than 0.05 (*p *= 0.047), this factor may only has a trend to increase the incidence of major complications after thermal ablation. More than half of the patients in our study developed within cirrhosis. As cirrhosis progressed, some patients' platelet counts were reduced due to hypersplenism, which could increase the risk of haemorrhage (30, 31). However, in our study, the incidence of haemorrhage was only 0.2% (4/2,084). Our previous study showed that with some preventive measures, thermal ablation is a safe method for patients with decreased PLT count (32).

SIRS is the body's excessive defensive stress response to pathogenic factors, which eventually transforms into a clinical syndrome in the pathological process of systemic inflammatory damage (33). For many years, SIRS was used to define sepsis. However, the concept of SIRS is too sensitive and lacks specificity, and the SIRS criterion is recognized to be limited as a prognostic tool in the general population (34). Thermal ablation can instantaneously induce massive production of necrotic tumour tissues and increase the risk of systemic inflammatory response, manifesting as elevated body temperature, leukocytosis, and increased C-reactive protein levels (35, 36), which are all assumed to participate in organ injuries such as hepatic abscesses and liver dysfunction. Thus, we included SIRS as a variable, and the results showed that it significantly increased the risk of major complications after thermal ablation. Therefore, close supervision of SIRS is expected to be necessary to implement interventional preventive measures as early as possible.

There are some limitations of this study, including its retrospective design and single treatment centre bias, which reduces the generalizability of our data. In addition, 35% of ablation sessions in this study were multifocal, and included more than two lesion locations, thus we did not analyse the impact of lesion location on complications in the study. Finally, since this study was a retrospective analysis, the power and frequency of thermal ablation could not be controlled, and there may have been selection biases.

## Conclusion

In conclusion, thermal ablation of liver tumours is a safe procedure with a low rate of major complications. A total maximum diameter of the lesions >3 cm, MWA and MWA + RFA utilization, and incidence of ICC and postoperative SIRS were the most statistically important risk factors for major complications. Reduced PLT counts (<100 × 10^9^/L) may have a tendency to increase the incidence of major complications. The factors revealed in this study will help to stratify high-risk patients.

## Data Availability

The original contributions presented in the study are included in the article, further inquiries can be directed to the corresponding author.

## References

[1] Bray F Ferlay J Soerjomataram I Siegel RL Torre LA Jemal A. Global cancer statistics 2018: GLOBOCAN estimates of incidence and mortality worldwide for 36 cancers in 185 countries. CA Cancer J Clin. (2018) 68:394–424. 10.3322/caac.21492 30207593

[2] Siegel RL Miller KD Jemal A. Cancer statistics, 2018. CA Cancer J Clin. (2018) 68:7–30. 10.3322/caac.21442 29313949

[3] EAFT L. EASL clinical practice guidelines: management of hepatocellular carcinoma. J Hepatol. (2018) 69:182–236. 10.1016/j.jhep.2018.03.019 29628281

[4] Heimbach JK Kulik LM Finn RS Sirlin CB Abecassis MM Roberts LR AASLD Guidelines for the treatment of hepatocellular carcinoma. Hepatology. (2018) 67:358–80. 10.1002/hep.29086 28130846

[5] Takahashi H Berber E. Role of thermal ablation in the management of colorectal liver metastasis. Hepatobiliary Surg Nutr. (2020) 9:49–58. 10.21037/hbsn.2019.06.08 32140478 PMC7026789

[6] Fang Y Chen W Liang X Li D Lou H Chen R Comparison of long-term effectiveness and complications of radiofrequency ablation with hepatectomy for small hepatocellular carcinoma. J Gastroenterol Hepatol. (2014) 29:193–200. 10.1111/jgh.12441 24224779

[7] Kutlu OC Chan JA Aloia TA Chun YS Kaseb AO Passot G Comparative effectiveness of first-line radiofrequency ablation versus surgical resection and transplantation for patients with early hepatocellular carcinoma. Cancer. (2017) 123:1817–27. 10.1002/cncr.30531 28085184

[8] Bertot LC Sato M Tateishi R Yoshida H Koike K. Mortality and complication rates of percutaneous ablative techniques for the treatment of liver tumors: a systematic review. Eur Radiol. (2011) 21:2584–96. 10.1007/s00330-011-2222-3 21858539

[9] Chen TM Huang PT Lin LF Tung JN. Major complications of ultrasound-guided percutaneous radiofrequency ablations for liver malignancies: single center experience. J Gastroenterol Hepatol. (2008) 23:e445–450. 10.1111/j.1440-1746.2007.05078.x 17683478

[10] Kasugai H Osaki Y Oka H Kudo M Seki T, Osaka Liver Cancer Study G. Severe complications of radiofrequency ablation therapy for hepatocellular carcinoma: an analysis of 3,891 ablations in 2,614 patients. Oncology. (2007) 72(Suppl 1):72–5. 10.1159/000111710 18087185

[11] Livraghi T Solbiati L Meloni MF Gazelle GS Halpern EF Goldberg SN. Treatment of focal liver tumors with percutaneous radio-frequency ablation: complications encountered in a multicenter study. Radiology. (2003) 226:441–51. 10.1148/radiol.2262012198 12563138

[12] Livraghi T Meloni F Solbiati L Zanus G. Complications of microwave ablation for liver tumors: results of a multicenter study. Cardiovasc Intervent Radiol. (2012) 35:868–74. 10.1007/s00270-011-0241-8 21833809

[13] Liang P Wang Y Yu X Dong B. Malignant liver tumors: treatment with percutaneous microwave ablation–complications among cohort of 1136 patients. Radiology. (2009) 251:933–40. 10.1148/radiol.2513081740 19304921

[14] Maeda M Saeki I Sakaida I Aikata H Araki Y Ogawa C Complications after radiofrequency ablation for hepatocellular carcinoma: a multicenter study involving 9,411 Japanese patients. Liver Cancer. (2020) 9:50–62. 10.1159/000502744 32071909 PMC7024979

[15] Fonseca AZ Santin S Gomes LG Waisberg J Ribeiro MJ Jr. Complications of radiofrequency ablation of hepatic tumors: frequency and risk factors. World J Hepatol. (2014) 6:107–13. 10.4254/wjh.v6.i3.107 24672640 PMC3959111

[16] Curley SA Marra P Beaty K Ellis LM Vauthey JN Abdalla EK Early and late complications after radiofrequency ablation of malignant liver tumors in 608 patients. Ann Surg. (2004) 239:450–8. 10.1097/01.sla.0000118373.31781.f2 15024305 PMC1356249

[17] Rhim H. Complications of radiofrequency ablation in hepatocellular carcinoma. Abdom Imaging. (2005) 30:409–18. 10.1007/s00261-004-0255-7 15688113

[18] Bruix J Sherman M, American Association for the Study of Liver D. Management of hepatocellular carcinoma: an update. Hepatology. (2011) 53:1020–2. 10.1002/hep.24199 21374666 PMC3084991

[19] Omary RA Bettmann MA Cardella JF Bakal CW Schwartzberg MS Sacks D Quality improvement guidelines for the reporting and archiving of interventional radiology procedures. J Vasc Interv Radiol. (2002) 13:879–81. 10.1016/S1051-0443(07)61769-2 12354820

[20] Ahmed M Solbiati L Brace C Breen D Callstrom M Charboneau J Image-guided tumor ablation: standardization of terminology and reporting criteria–a 10-year update. Radiology. (2014) 273:241–60. 10.1148/radiol.14132958 24927329 PMC4263618

[21] de Baere T Risse O Kuoch V Dromain C Sengel C Smayra T Adverse events during radiofrequency treatment of 582 hepatic tumors. AJR Am J Roentgenol. (2003) 181:695–700. 10.2214/ajr.181.3.1810695 12933462

[22] Xu E Li K Long Y Luo L Zeng Q Tan L Intra-procedural CT/MR-ultrasound fusion imaging helps to improve outcomes of thermal ablation for hepatocellular carcinoma: results in 502 nodules. Ultraschall Med. (2021) 42(2):e9–e19. 10.1055/a-1021-1616: 31671457

[23] Head HW Dodd GD 3rd Dalrymple NC Prasad SR El-Merhi FM Freckleton MW Percutaneous radiofrequency ablation of hepatic tumors against the diaphragm: frequency of diaphragmatic injury. Radiology. (2007) 243:877–84. 10.1148/radiol.2433060157 17517940

[24] Kim YK Kim CS Lee JM Chung GH Chon SB. Efficacy and safety of radiofrequency ablation of hepatocellular carcinoma in the hepatic dome with the CT-guided extrathoracic transhepatic approach. Eur J Radiol. (2006) 60:100–7. 10.1016/j.ejrad.2006.05.002 16781835

[25] Kang TW Rhim H Kim EY Kim YS Choi D Lee WJ Percutaneous radiofrequency ablation for the hepatocellular carcinoma abutting the diaphragm: assessment of safety and therapeutic efficacy. Korean J Radiol. (2009) 10:34–42. 10.3348/kjr.2009.10.1.34 19182501 PMC2647171

[26] Dodd GD 3rd Frank MS Aribandi M Chopra S Chintapalli KN. Radiofrequency thermal ablation: computer analysis of the size of the thermal injury created by overlapping ablations. AJR Am J Roentgenol. (2001) 177:777–82. 10.2214/ajr.177.4.1770777 11566672

[27] Liang P Wang Y. Microwave ablation of hepatocellular carcinoma. Oncology. (2007) 72(Suppl 1):124–31. 10.1159/000111718 18087193

[28] Giorgio A Gatti P Montesarchio L Santoro B Dell'Olio A Crucinio N Intrahepatic cholangiocarcinoma and thermal ablation: long-term results of an Italian retrospective multicenter study. J Clin Transl Hepatol. (2019) 7:287–92. 10.14218/JCTH.2019.00036 31915596 PMC6943218

[29] Su XF Li N Chen XF Zhang L Yan M. Incidence and risk factors for liver abscess after thermal ablation of liver neoplasm. Hepat Mon. (2016) 16:e34588. 10.5812/hepatmon.34588 27642345 PMC5018304

[30] Under the auspices of the Italian Association for the Study of Liver Diseases (AISF) and the Italian Society of Internal Medicine (SIMI). Hemostatic balance in patients with liver cirrhosis: report of a consensus conference. Dig Liver Dis. (2016) 48:455–67. 10.1016/j.dld.2016.02.008 27012444

[31] Basili S Raparelli V Violi F. The coagulopathy of chronic liver disease: is there a causal relationship with bleeding? Yes. Eur J Intern Med. (2010) 21:62–4. 10.1016/j.ejim.2010.01.005 20206871

[32] Huang Q Xu E Tan L Zeng Q Zheng R Li K. Thermal ablation of hepatocellular carcinoma in patients with abnormal coagulation function. Int J Hyperthermia. (2018) 34:1038–43. 10.1080/02656736.2017.1390787 29082796

[33] Margraf A Ludwig N Zarbock A Rossaint J. Systemic inflammatory response syndrome after surgery: mechanisms and protection. Anesth Analg. (2020) 131:1693–707. 10.1213/ANE.0000000000005175 33186158

[34] Kaukonen KM Bailey M Pilcher D Cooper DJ Bellomo R. Systemic inflammatory response syndrome criteria in defining severe sepsis. N Engl J Med. (2015) 372:1629–38. 10.1056/NEJMoa1415236 25776936

[35] Jansen MC van Wanrooy S van Hillegersberg R Rijken AM van Coevorden F Prevoo W Assessment of systemic inflammatory response (SIR) in patients undergoing radiofrequency ablation or partial liver resection for liver tumors. Eur J Surg Oncol. (2008) 34:662–7. 10.1016/j.ejso.2007.06.009 17892922

[36] Bulvik BE Rozenblum N Gourevich S Ahmed M Andriyanov AV Galun E Irreversible electroporation vs. radiofrequency ablation: a comparison of local and systemic effects in a small-animal model. Radiology. (2016) 280:413–24. 10.1148/radiol.2015151166 27429143

